# Fusion of PCA and ICA in Statistical Subset Analysis for Speech Emotion Recognition

**DOI:** 10.3390/s24175704

**Published:** 2024-09-02

**Authors:** Rafael Kingeski, Elisa Henning, Aleksander S. Paterno

**Affiliations:** 1 Center for Science and Technology, Department of Electrical Engineering, Santa Catarina State University (UDESC), Joinville 89219-710, SC, Brazil; 2 Center of Technological Sciences, Department of Mathematics, Santa Catarina State University (UDESC), Rua Paulo Malschitzki, 200, Zona Industrial Norte, Joinville 89219-710, SC, Brazil; elisa.henning@udesc.br

**Keywords:** speech emotion recognition, feature selection, PCA, ICA, SVM, Kruskal–Wallis

## Abstract

Speech emotion recognition is key to many fields, including human–computer interaction, healthcare, and intelligent assistance. While acoustic features extracted from human speech are essential for this task, not all of them contribute to emotion recognition effectively. Thus, reduced numbers of features are required within successful emotion recognition models. This work aimed to investigate whether splitting the features into two subsets based on their distribution and then applying commonly used feature reduction methods would impact accuracy. Filter reduction was employed using the Kruskal–Wallis test, followed by principal component analysis (PCA) and independent component analysis (ICA). A set of features was investigated to determine whether the indiscriminate use of parametric feature reduction techniques affects the accuracy of emotion recognition. For this investigation, data from three databases—Berlin EmoDB, SAVEE, and RAVDES—were organized into subsets according to their distribution in applying both PCA and ICA. The results showed a reduction from 6373 features to 170 for the Berlin EmoDB database with an accuracy of 84.3%; a final size of 130 features for SAVEE, with a corresponding accuracy of 75.4%; and 150 features for RAVDESS, with an accuracy of 59.9%.

## 1. Introduction

Emotion detection systems aim to identify emotional states based on physiological signals, including voice signals as an important indicator [1]. In addition, emotion recognition has been applied extensively, including to the detection of health conditions (e.g., depression) [2,3], the synthesis of emotionally expressive speech (which is particularly relevant in virtual reality to enhancing the user experience) [4], and the assessment of service quality across various contexts [5,6]. With computational processing techniques, speech emotion recognition (SER) systems have been developed using acoustic features extracted from toolkits such as PRAAT [7] openSMILE [8], and VOICEBOX [9], as shown in [10]. These toolkits provide diverse features, some of which may be redundant or irrelevant for emotion recognition tasks. Selecting informative features is essential to enhance the performance of SER systems and optimize computational efficiency, and previous research has explored the use of various techniques for this purpose, such as principal component analysis (PCA) [11,12,13,14], independent component analysis (ICA) [15,16], statistical feature selection [14], and meta-heuristic algorithms [17,18]. These studies highlight the need to categorize processing techniques or develop a protocol to facilitate the integration of the most appropriate strategies for improving accuracy.

The purpose of our work was to investigate whether splitting the features into two subsets by distribution and applying commonly used feature reduction methods would impact accuracy, with emphasis on this protocol having the potential to improve upon the existing methods. In particular, our improvements were demonstrated using a standard reference method for emotion classification, which was applied to the input parameters using support vector machines. This research investigated how statistical analysis and initial feature characterization based on distribution can improve the process of selecting feature reduction techniques. Following a thorough statistical analysis with principal component analysis (PCA) and independent component analysis (ICA), we reorganized the vocal features into distinct groups based on their distribution characteristics, dividing them into normal and non-normal distributions, which facilitated the targeted application of PCA and ICA.

The remainder of this paper is structured as follows: Section 2 provides a description of related works. Section 3 includes a description of the databases, mathematical notation and certain definitions, the proposed method, the feature selection, and the reduction techniques. Section 4 presents the results obtained through the application of the method proposed. Section 5 discusses the results. Section 6 concludes and outlines future work.

## 2. Related Works

According to [17], computational speech emotion recognition can be performed using the following four steps: (1) voice signal pre-processing, (2) the extraction of relevant features, (3) the selection and fusion of these features, and (4) emotion classification. Several studies have focused on specific aspects of this process, such as feature extraction optimization [1,19], feature reduction [20], and classifier selection as most suits the task [13,21]. In this research, the focus was on feature selection and reduction.

On the basis that they contain significant information for classification, paralinguistic features are commonly extracted [1,14,17]. One study [1] reviewed previous research that employed vocal features and evaluated their emotional impact. Additionally, paralinguistic acoustic features have been used widely for emotion detection [17,18,21,22]. These features serve as inputs for classifiers, which are mathematical models developed from the data available to identify emotions in other datasets. However, not all the features extracted from speech audio are useful for increasing model accuracy. To the best of our knowledge, feature selection optimization methods have not been applied within such investigations yet, although some authors have opted to use linear reduction methods such as PCA and ICA. In this work, PCA and ICA were selected, as PCA is often used without prior feature selection, thus disregarding the data distribution, while ICA is a non-parametric method that is recommended when the data are not normally distributed [23].

While PCA yields optimal results when the data are normally distributed [23], it remains applicable even when the data are non-Gaussian [23]. On the other hand, independent component analysis (ICA) is a technique used to identify underlying factors or components in multivariate statistical datasets and find statistically independent and non-Gaussian components [24]. Certain researchers have used ICA to recognize the emotions in vocal features, albeit less frequently [15,16].

We can see one example of research focusing on this type of analysis in [25], in which the data distribution was analyzed by separating them into normal and non-normal sets. However, it did not propose reducing the features based on the distribution criteria. Other studies have considered the data distribution and applied feature reduction techniques using ICA and PCA, for example, within chemical process monitoring [26,27], but it is noteworthy that these proposed methods have not yet been applied to the problem of speech emotion recognition.

## 3. Materials and Methods

This section outlines the databases used; the feature extraction tool; the methods for feature selection, reduction, and component fusion; and, finally, the classifier used to validate the proposed technique. As described in the previous section, emotion recognition can be divided into four parts; however, the focus of this research was solely on one of these parts: the selection of and a reduction in features.

### 3.1. The Speech Database

As presented in Table 1, the databases used included the Berlin database (EmoDB), which comprised recordings from 10 actors, 5 male and 5 female, and emotional expression spanning anger, fear, boredom, disgust, happiness, sadness, and neutral states [28]; the SAVEE dataset, consisting of voice recordings from 4 male actors across 7 distinct emotions for a total of 480 utterances in British English [29]; and RAVDESS, featuring 24 professional actors, each contributing 104 unique vocalizations in English that covered emotions such as happiness, sadness, anger, fear, surprise, disgust, calmness, and neutrality [30].

These databases were selected due to their accessibility and widespread usage, with previous applications within similar studies [14,17].

### 3.2. Mathematical Notation

To better describe the notations and acronyms used in this work, Table 2 outlines their meanings. This was conducted to support a better understanding of the equations and algorithms presented in this article.

### 3.3. The Proposed Method

**Figure 1:**
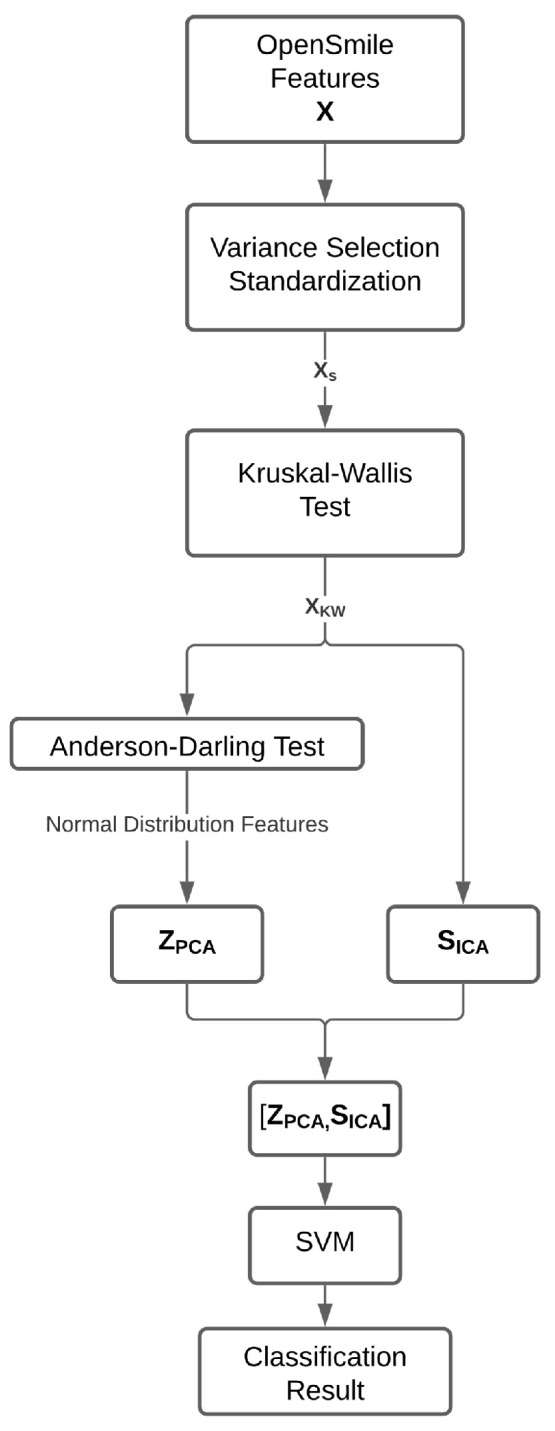
Flow diagram of the proposed method. Filtering achieved via variance and the Kruskal–Wallis test, which was then split using the Anderson–Darling test; then, PCA and ICA fusion was utilized for SVM classification.

The proposed method was divided into steps, as shown in Figure 1. After filtering, the data were split into a normal subset for PCA, ICA, and PCA and ICA fusion, which were then used as the input for the SVM for speech emotion recognition. The corresponding pseudocode is shown in Algorithm 1. Each step of the proposed method is described in this section, with each step detailed in its own subsection.

Combining PCA and ICA leverages the strengths of both methods. PCA is applied to reduce the features, especially when dealing with high-dimensional datasets [23]. This step ensures that the primary variance in the data is captured. Then, ICA is applied to the data with reduced dimensions to extract independent components that might reveal additional underlying structures not captured using PCA alone.
**Algorithm 1:** Feature extraction and selection for SER.**Input:** Audio files and corresponding emotion labels **Output:** Optimized feature sets for SER
 1:Step 1: Load and pre-process the database 2:db ← LoadDatabase(‘database_name’) 3:Y ← emotions_labels 4:Step 2: Extract features using openSMILE 5:X ← smile.process_files(db) 6:Step 3: Filter quasi-constant features 7:X_norm_ ← NormalizeFeatures(X) 8:X_fnorm_ ←FilterQuasiConstants(X_norm_, threshold = $10^{-5}$) 9:Step 4: Standardize the features10:X_s_ ← StandardizeFeatures(X,X_fnorm_)11:Step 5: Select features with the Kruskal–Wallis test12:X_kw_ ← KruskalWallisTest(X_s_)13:Step 6: Test the distribution of the features14:X_n_, X_nn_ ← TestDistribution(feats_df, 95)15:Step 7: Apply PCA and ICA transformations16:X_PCA_ ← PCATransformation(X_n_, n_components = 100)17:**for** i←10 **to** 100 **step** 10 **do**18:   X_ica_[i] ← ICATransformation(X_kw_)19:**end for**20:Step 8: Evaluate the models using cross-validation21:X_input_ ← concat(X_pca_, X_ica_)22:result_matrix ← CrossValidation(X_input_, Y, SVM_model)


By combining PCA and ICA, both the principal variance (via PCA) and the independent, non-normal distribution (via ICA) in the data are captured, which is advantageous when dealing with complex datasets that contain both normal and non-normal features.

Although more accurate emotion classification was not the primary focus of this research, the multi-stage feature selection method proposed aimed to improve the process by filtering out data that were less informative. This filtration process was designed to clean the input data for PCA and ICA, ensuring that only relevant and informative features were subsequently analyzed. The stages included initial selection by low variance, followed by the Kruskal–Wallis test, as used in [31]. All scripts were developed in Python 3.12 [32] and are available in the following https://github.com/rkingeski/pca_ica_speech_emotion, accessed on 18 June 2024).

### 3.4. Feature Extraction

For feature extraction, we utilized the openSMILE toolkit 3.0.2 [8], an open-source Python library that provides various feature groups for selection. In this work, the ComPare 2013 group was chosen, comprising 6373 features derived by combining 64 low-level descriptors such as energy, mel-frequency cepstral coefficients (MFCCs), and pitch, which were then applied to functional descriptors that included the mean, minimum, maximum, standard deviation, and so on. For more details, see [8,33].

To mathematically represent the data, a matrix X with the dimensions m×n was considered, where $x_{ij}$ represents the element in Row *i* and Column *j*. The rows correspond to the recordings, and the columns relate to individual features across all recordings, where *m* denotes the number of recordings that varied for each dataset and *n* denotes the number of features extracted (which, in this case, was 6373). Additionally, a class vector Y of size *m* was defined, where each element represents an emotion. A type of supervised machine learning model was used due to the availability of class labels for classification.

The data organization is illustrated in Equation (1), where the classes and features are juxtaposed as follows:

(1) Data=[YX].

In Equation (1), each $Y_{i}$ corresponds to $X_{j}$ acoustic features, as described in this section.

### 3.5. Variance Feature Selection

Preliminary variance-based selection was performed before applying the features to PCA and ICA, aiming to eliminate constant features and those with particularly low variance. Removing constant features is important since they do not contribute to the model and, hence, do not provide any information relevant to the analysis. Additionally, features with low variance may only provide a relatively minor contribution to the model, potentially skewing the representation of the data. Therefore, by removing features with low variance, we aim to ensure a more significant and informative representation of the data. This initial filtering step serves as a pre-selection that can enhance the performance of feature reduction algorithms such as PCA and ICA.

Although variance is an important indicator of information, it is not the sole criterion, and the amount of information does not guarantee its usefulness in discriminating between classes [34]. The variance in the data was calculated according to Equation (2), where $\bar{x}$ denotes the mean of the column values. For each column, the variance was computed after normalizing matrix X to a range between 0 and 1. *m* represents the number of audio records or the number of lines in matrix $X_{\mathrm{norm}}$, and ${\bar{x}}_{j}$ represents the mean calculated for each row *j*.

(2) $$\mathrm{Var}\left(X_{\mathrm{norm}}\right)=\frac{1}{m}\sum_{i=1}^{m}{\left(x_{ij}-{\bar{x}}_{j}\right)}^{2}.$$

In Equation (2), $x_{ij}$ represents the element in Row *i* and Column *j* in the normalized matrix $X_{\mathrm{norm}}$.

For feature reduction and a more focused analysis, columns from $X_{\mathrm{norm}}$ were selected based on their variance using an arbitrary cutoff value of $1\times 10^{-5}$ as the minimum acceptable variance, which was opted for on the basis of the preliminary data analysis, aiming to achieve a balance between retaining important information and removing features with low variance. The selection of $1\times 10^{-5}$ was considered appropriate as it resulted in the removal of less than 10% of the features across all the datasets used in this research, thereby achieving feature reduction, as shown in Table 3.

Data exhibiting higher variance were standardized to a mean of (μ=0) and a standard deviation of (σ=1). The standardized data matrix can be represented using Equations (3) and (4), as follows:

(3) $$x_{ij}^{\prime }=\frac{x_{ij}-{\bar{x}}_{i}}{\sigma_{j}},$$

where

(4) $$X_{s}=\left[\begin{array}{cccc} x_{11}^{\prime } & x_{12}^{\prime } & \cdots & x_{1k}^{\prime }\\ x_{21}^{\prime } & x_{22}^{\prime } & \cdots & x_{2k}^{\prime }\\ \vdots & \vdots & \ddots & \vdots \\ x_{m1}^{\prime } & x_{m2}^{\prime } & \cdots & x_{mk}^{\prime } \end{array}\right].$$

The dimensions of $X_{S}$ are m×k, where *m* is the number of input audio records, and *k* is the number of features selected by variance. The specific values for each database are detailed in Table 4.

### 3.6. Kruskal–Wallis Feature Selection

Following initial variance-based feature selection, additional selection using the Kruskal–Wallis test is recommended. This non-parametric test, akin to ANOVA, is employed when the data distribution is unknown to evaluate whether it is statistically equivalent among different groups [35]. In this research, emotions were treated as the response classes, and the data distribution across these classes was examined for each feature.

Regarding the Kruskal–Wallis test, the null hypothesis was rejected for each feature that showed statistical evidence of distributional differences among groups, with a significance level of 1% utilized to minimize the number of features selected. Consequently, these features were deemed critical for the model, as rejection of the null hypothesis suggests that at least one group exhibits a statistically distinct distribution compared with the others.

While the test identifies features with statistically different distributions across groups, it does not pinpoint which specific class displays the distinct distribution. Therefore, a new subset was defined by Equation (5), from which features lacking statistical differences were excluded.

(5) $$X_{\mathrm{KW}}=\left\{x_{j}|x_{j}\in X_{s},\;p\text{-value}\left(x_{j}\right)\leq \alpha \right\}.$$

$X_{\mathrm{KW}}$ represents the features filtered through the Kruskal–Wallis test. The number of features for each database after filtering is shown in Table 4.

### 3.7. The Anderson–Darling Test

To assess the adequacy of the models for the data observed, we employed the Anderson–Darling test, which is a statistical method used to check whether a data sample follows a normal distribution [36]. A significance level of 0.05 was adopted as the *p*-value criterion and the test applied to the data matrix $X_{\mathrm{KW}}$, analyzing each of its columns individually. After their separation, the two groups of data were represented with Equation (6), as follows:

(6) $$X_{\mathrm{KW}}=\left[X_{n}\;X_{\mathrm{nn}}\right],$$

where $X_{n}$ represents the subset of normally distributed data and $X_{\mathrm{nn}}$ represents the subset of non-normal data. The dimensions are provided in Table 3 under the columns labeled Normal KW and Non-Normal KW.

### 3.8. Principal Component Analysis

Principal component analysis (PCA) is a commonly employed feature reduction technique that projects features onto a new basis that captures the maximum data variance [34]. However, in this context, the goal was to analyze the feature distribution, so as PCA was not deemed ideal for this specific case, fusion analysis was applied. Furthermore, combining both methods was hoped to potentially enhance the classifier performance.

The principal components were calculated with Equation (7), as follows:

(7) $$Z_{\mathrm{PCA}}=W_{P}X_{n},$$

where $W_{P}$ is the matrix of eigenvectors corresponding to the largest eigenvalues of the covariance matrix of $X_{n}$.

Using PCA, the data were transformed into new variables that linearly combined the original ones. As the goal was to find the highest variance, we can assume an input signal in this work as $X_{n}$ with dimensions of m×l, *m*, which are defined as the number of input audio records, where *l* is the number of features with a normal distribution.

### 3.9. Independent Component Analysis

Independent component analysis (ICA)—a technique developed to separate signals generated by independent sources and initially proposed to solve the problem of blind source separation (BSS)—can separate linearly combined signals. It is a non-parametric method that can identify the original components that compose the signals observed, even when the combinations of these components are complex; however, it does not require distribution-based feature separation [37].

In this context, we do not directly deal with signals but rather features, which will serve as the input to the classifier. We applied ICA to separate the underlying independent components based on the premise that these features represent combinations of components and reduce the number of input features while retaining important information for classification. This approach seeks to enhance the classifier’s ability to recognize distinct patterns by assuming the virtual separation of features into independent components.

The ICA model can be described as follows: considering the given features as independent signals $S_{\mathrm{ICA}}=\left[s_{1,ica}\;s_{2,ica}\;s_{3,ica}\;\ldots \;s_{i,ica}\right]$, if and only if they are independent and have a non-normal distribution, when they are mixed (possibly with only one normally distributed component incorporated), then new signals are created such that $X_{\mathrm{KW}}=\left[x_{kw1}\;x_{kw2}\;x_{kw3}\;\ldots \;x_{kwi}\right]$, which is a combination of the features $S_{\mathrm{ICA}}$ [37]. We can describe the combination in question with Equation (8), as follows:

(8) $$X_{\mathrm{KW}}=AS_{\mathrm{ICA}},$$

where A is the mixing matrix of the independent signals or, in this case, the mixed features.

Under the conditions of the signals’ independence, we can estimate a matrix W that solves the system and recovers the signals, which is described in the following Equation (9).

(9) $$S_{\mathrm{ICA}}=WX_{\mathrm{KW}}.$$

In this case, let us assume we have an unknown source, represented by $S_{\mathrm{ICA}}$, which, when mixed, results in the voice features $X_{\mathrm{KW}}$. There is no direct relationship in this mixture; rather, we hypothesize that there are a combination of values in $S_{\mathrm{ICA}}$ that result in $X_{\mathrm{KW}}$. Therefore, upon separating the features, we obtain new data, represented by $S_{\mathrm{ICA}}$.

We applied the FastICA algorithm, which was implemented in the Scikit-learn library. The algorithm was configured according to the theoretical guidelines, and the specific details of its configuration are described in Table 5.

### 3.10. The Support Vector Machine

Support vector machine (SVM) classifiers are based on the separation of data groups via hyperplanes. The general idea is to map the input parameters to a higher-order space in a non-linear fashion and then to subsequently use hyperplanes to separate the data in this new space [38]. They have been frequently employed in research on voice emotion recognition [14,17,21,39]. In this research, we chose to use this model since the focus was not selecting the most suitable algorithm for model generation.

For the models generated in this work, a radial basis function was adopted for the kernel in support vector classification (SVC). Initially, the kernel tuning parameters used for SVC were the default parameters from the Scikit-learn library [40]. Subsequently, adjustments were then made to the model parameters *C* and γ. The parameter γ was set as the default, as represented in Equation (10), where *n* represents the number of input data points and $\sigma_{X}$ denotes the variance in the input data. Values for C of 0.1, 1, 10, 100, and 1000 were tested, with a final value of 100 chosen.

(10) $$\gamma =\frac{1}{\left(n\cdot \sigma_{X}\right)}.$$

The models were employed using pre-existing datasets previously described in the literature to test and validate the feature selection method proposed in this article, as shown in Table 1. Accuracy was evaluated using the cross-validation method with k=10, as suggested by [41].

### 3.11. Metrics

In this research, four metrics were used to evaluate the performance of the models. The average class accuracy of a classifier, or the mean accuracy (given by Equation (11)), is a metric that represents the number of correct predictions made by a model. Precision, which is given by Equation (12), is the average agreement per class of the data class labels with those of the classifier. Recall, which is given by Equation (13), is the average of the classifier’s effectiveness in identifying class labels. The F-score, which is given by Equation (14), is the harmonic mean of the precision and recall [42].

(11) $$\mathrm{Accuracy}=\frac{\sum_{i=1}^{l}{\mathrm{tp}}_{i}+{\mathrm{tn}}_{i}}{\sum_{i=1}^{l}\left({\mathrm{tp}}_{i}+{\mathrm{fn}}_{i}+{\mathrm{fp}}_{i}+{\mathrm{tn}}_{i}\right)},$$

(12) $$\mathrm{Precision}=\frac{\sum_{i=1}^{l}{\mathrm{tp}}_{i}}{\sum_{i=1}^{l}\left({\mathrm{tp}}_{i}+{\mathrm{fp}}_{i}\right)},$$

(13) $$\mathrm{Recall}=\frac{\sum_{i=1}^{l}{\mathrm{tp}}_{i}}{\sum_{i=1}^{l}\left({\mathrm{tp}}_{i}+{\mathrm{fn}}_{i}\right)},$$

(14) $$\text{F-score}=\frac{(b^{2}+1)\times \mathrm{Precision}\times \mathrm{Recall}}{b^{2}\times \mathrm{Precision}+\mathrm{Recall}},$$

where ${\mathrm{tp}}_{i}$ represents the true positives, ${\mathrm{fp}}_{i}$ represents the false positives, ${\mathrm{fn}}_{i}$ represents the false negatives, and ${\mathrm{tn}}_{i}$ represents the true negatives for the *i*-th class. The constant *b* is a weighting factor, and in this study, *b* was set to 1.

## 4. Results

Initially, 6373 features were extracted using the openSMILE toolkit. After filtering out features with low variance, the Kruskal–Wallis test was applied to aid in identifying and removing features that did not show statistically significant differences among the classes [35]. This test verified whether there was at least one emotion for which the individually tested features exhibited a distribution difference when compared with the other emotions. Features that did not show differences were discarded, thus forming a subset that was further divided into two subsets after the Anderson–Darling test: features with a normal distribution and features with a non-normal distribution.

To compare the results, accuracy was tested using 100 principal components and 100 independent components in the following two ways: (1) applying PCA and ICA to all the features, that is, 4382, 4845, and 5407 features for the SAVEE, RAVDESS, and Berlin EmoDB databases, respectively, and (2) applying ICA to all the features and applying PCA only to the normally distributed features, thus resulting in totals of 415, 133, and 397 for the SAVEE, RAVDESS, and Berlin EmoDB databases, respectively. The number of features with normal and non-normal distributions, along with the features selected by variance, is presented in Table 3.

**Figure 2:**
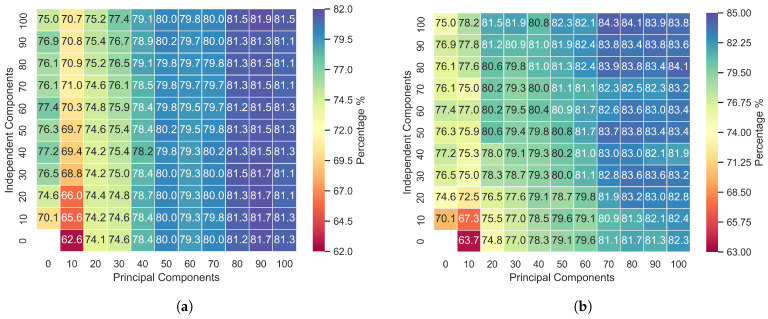
Accuracy of the PCA and ICA fusion for the EmoDB database: (**a**) PCA applied to all features and (**b**) PCA applied to features with a Gaussian distribution.

**Figure 3:**
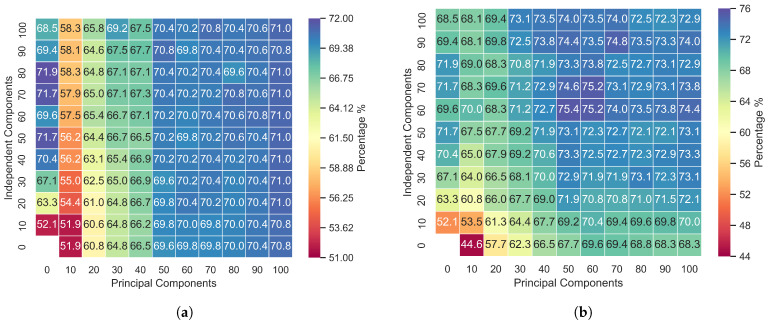
Accuracy of the PCA and ICA fusion for the SAVEE database: (**a**) PCA applied to all features and (**b**) PCA applied to features with a Gaussian distribution.

**Figure 4:**
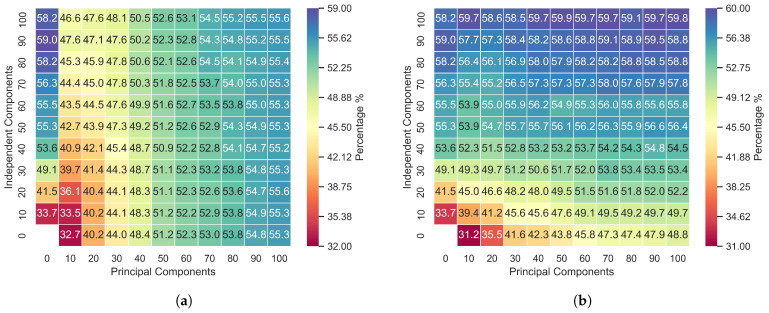
Accuracy of the PCA and ICA fusion for the RAVDESS database: (**a**) PCA applied to all features and (**b**) PCA applied to features with a Gaussian distribution.

In Figure 2, Figure 3 and Figure 4, the first column from left to right represents the first 100 independently calculated components. This procedure was carried out assuming that the input features could be decomposed into 10 components, 20 components, 30 components, etc., up to 100 components. The top first row of the graphs in Figure 2, Figure 3 and Figure 4 displays the principal components ordered by highest variance. The other points are concatenations of PCA and ICA.

Figure 2a, Figure 3a and Figure 4a represent the accuracy when the data were not segmented by distribution. In these cases, principal component analysis (PCA) and independent component analysis (ICA) techniques were applied to all the data selected via the Kruskal–Wallis test, and then the results were combined as per the diagram. In Figure 2b, Figure 3b and Figure 4b, the principal components were calculated exclusively from acoustic features that exhibited a normal distribution according to the Anderson–Darling test.

**Figure 5:**
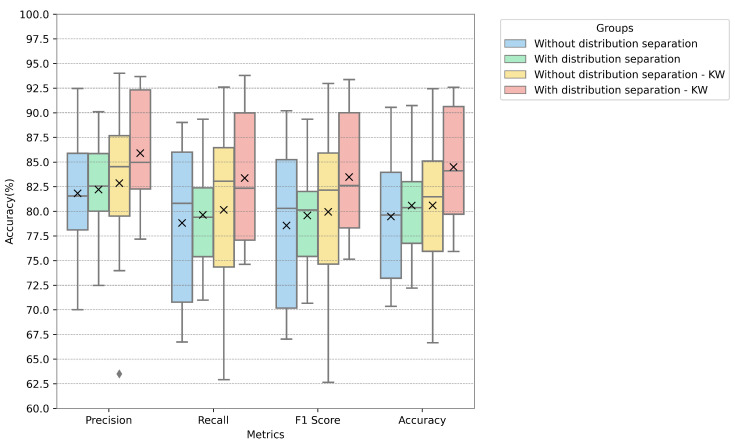
Metrics for the EmoDB database when using 100 independent components and 70 principal components.

**Figure 6:**
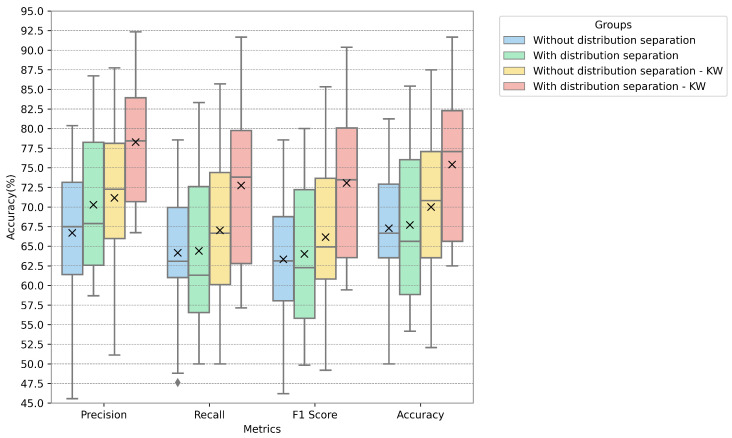
Metrics for the SAVEE database when using 60 independent components and 50 principal components.

**Figure 7:**
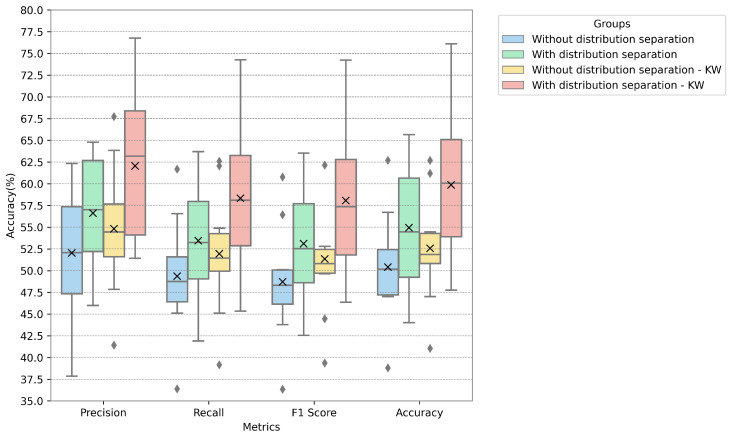
Metrics for the RAVDESS database using 100 independent components and 50 principal components.

In Figure 5, Figure 6 and Figure 7, the metrics for each database are presented with the distribution, median, and mean values. The metrics used in these figures are described by Equations (11)–(14), representing accuracy, precision, recall, and F-score. We fused PCA and ICA using the highest value for the proposed techniques (i.e., using the PCA calculated only on normally distributed data and the ICA calculated on all features). The values of the groups were both separated according to the legend by normal distribution and left unseparated, with the Kruskal–Wallis test also applied for feature reduction.

**Figure 8:**
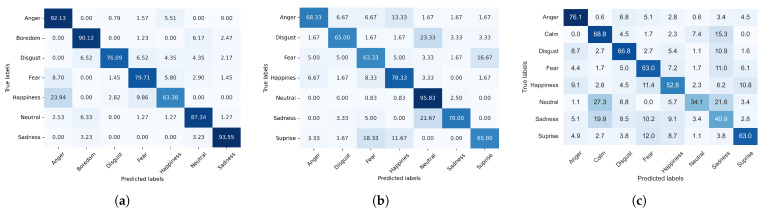
Confusion matrix for the (**a**) EmoDB, (**b**) SAVEE, and (**c**) RAVDESS databases.

Finally, to understand the results in each database better, confusion matrix plots were generated, as shown in Figure 8.

## 5. Discussion

This research explored techniques for feature selection and reduction. The number of input features was reduced by dividing them into two subsets based on distribution before applying PCA and ICA. Specifically, PCA and ICA allowed us to identify and select the most informative features, thus enhancing the accuracy by focusing on these key features while discarding less pivotal ones, thereby reducing the number of parameters used in the classifier.

PCA was used to reduce the features by capturing the principal variance for the normal distribution subset, while ICA was used to extract the data from both the normal and non-normal datasets, thus assuming a combination of features.

Applying PCA and ICA to all the features without discriminating by distribution revealed that the principal components have a greater influence on the model, as evidenced in Figure 2a, Figure 3a and Figure 4a. Concatenating the two feature groups ($S_{\mathrm{ICA}}$,$Z_{\mathrm{PCA}}$) without separation based on the distribution resulted in minimal differences in the accuracy due to the independent components, with the RAVDESS dataset showing slightly more influence. Notably, this dataset has the fewest features with a normal distribution, as indicated in Table 3 and Table 4.

The proposed model achieved the highest accuracy with the EmoDB dataset, followed by SAVEE and RAVDESS. With EmoDB, the accuracy improved from 80.0% to 84.3% when using 100 independent components and 70 principal components, which represented the highest accuracy, as shown in Figure 2. When comparing the accuracy values between the principal and independent components for EmoDB (the first row and column in Figure 2a and Figure 2b, respectively), using only 397 features with a normal distribution (see Table 3) and 100 principal components resulted in improved accuracy. This suggests that components with a normal distribution significantly contribute to the model’s performance, and applying PCA solely to features with a normal distribution enhances the accuracy; however, the best performance was achieved with PCA+ICA. For SAVEE, the most significant reduction in the number of components and improvement in accuracy were observed, with the accuracy increasing from 70.0% to 75.4% using a combination of 60 independent and 50 principal components (with a normal distribution). SAVEE had the highest number of normally distributed features, as shown in Table 3. Notably, the proposed method led not only to a higher mean and median accuracy but also to an improvement in the upper quartile within the same component group (with 50 principal components and 60 independent components), as is visible in Figure 6.

RAVDESS exhibited the lowest performance using the method proposed, likely due to it containing the fewest normally distributed features. Additionally, using ICA alone yielded better accuracy than PCA, as depicted in Figure 4, thus further indicating that PCA is less performant for non-normally distributed data. The metrics in Figure 7 indicate the model’s improvement, where the accuracy increased from 52.6% to 59.9% for 100 independent components and 50 principal components.

In this research, confusion matrices (Figure 8) were used to evaluate the performance of the proposed model for each emotion after selecting the best PCA+ICA results separated by distribution. In Figure 8a, the confusion matrix shows that happiness and anger were confused for one another more often for the EmoDB dataset. For the SAVEE dataset (Figure 8b), the highest accuracy was observed for a neutral state, possibly due to the dataset’s imbalance, in that 120 samples covered neutrality, compared with only 60 samples covering the other classes. For the RAVDESS dataset (Figure 8c), three classes were confused for one another most frequently: neutral, calm, and sadness. Improving the average accuracy here may be facilitated by discarding one class, such as the calm class, which is less prevalent within the datasets in the literature and may negatively impact recognition systems applying the method proposed in this research.

Finally, we compared the results obtained in this research with those of other works that have used PCA or combined PCA with another feature reduction technique, as shown in Table 6. The results of this research had an accuracy value consistent with prior research, albeit the model proposed here performed better in all cases. We also compared the proposed model with other speech emotion recognition techniques, as shown in Table 7.

In order to contextualize the comparison of the results from this research with those from other research, it is important to highlight that some other studies did not utilize the same 6373 acoustic features from the openSMILE library employed in this research. Additionally, the validation methods that were adopted differed between different studies. While we employed 10-fold cross-validation, other works used methods such as an 80–20 data split or leave-one-speaker-out, as shown in Table 6 and Table 7, which may have influenced the accuracy of the results.

The primary goal of this work was not to surpass the accuracy achieved by the state-of-the-art methods but rather to propose a novel approach that considers the feature distribution when applying feature reduction methods such as principal component analysis (PCA) and independent component analysis (ICA).

The results of this research demonstrate that the multi-stage feature selection method we have proposed achieves better accuracy than similar methods; see Table 6. We ensured that only the most relevant and informative features were utilized by filtering out uninformative data before applying PCA and ICA, thus leading to improvements in the model’s performance.

## 6. Conclusions and Final Considerations

In this research, a feature selection and reduction method was proposed utilizing subsets that considered the distribution of the acoustic voice features, with PCA and ICA applied to improve the accuracy of detecting emotions.

It was observed that creating two distinct subsets—one for features with a normal distribution and another for features with a non-normal distribution—and subsequently applying PCA to the normal features and ICA to all the features resulted in increased accuracy and reduced the number of features required. This method was validated on three distinct databases: Berlin EmoDB, SAVEE, and RAVDESS.

The results in Figure 2, Figure 3 and Figure 4 show that we successively achieved the objective of this research. In splitting the data into two subsets and applying PCA to the normally distributed subset and ICA to the entire dataset, the method proposed clearly demonstrates that PCA affects the results when it is applied exclusively to normally distributed features. Additionally, incorporating ICA and combining it with PCA further enhances the model’s performance, showing that ICA is a good alternative when handling non-normally distributed data.

The results show that the model exhibited the highest overall accuracy for the EmoDB database, particularly when only using PCA on normally distributed features, for which 82.3% accuracy was achieved using 100 principal components, as seen in Figure 2b. This highlights the importance of features with a normal distribution in constructing models using principal component analysis.

The accuracy for the SAVEE database was improved with the method proposed, with a 5% increase in accuracy for the same number of components. On the other hand, the proposed method performed poorly when it was applied to the RAVDESS database, which contained the lowest number of normally distributed features. Comparing the accuracy for 90 independent components with that for 100 independent components, more than 50 principal components showed an increase of only 0.9%, emphasizing the relationship between the efficacy of PCA and the number of features with a normal distribution.

Additionally, the confusion matrices revealed specific patterns of confusing certain emotions, indicating areas where the model could be enhanced. Comparison with the related literature demonstrates that the method proposed is competitive, achieving a similar or superior accuracy using fewer input features and therefore requiring a lower computational cost.

In considering the data distribution and applying PCA and ICA differently, the subset method proposed proved effective in improving the accuracy of emotion classification and reducing the number of features input into the model. This result underscores the importance of analyzing the feature distribution before selecting a reduction method in demonstrating that applying a selection method before PCA and ICA enhanced the proposed method’s accuracy.

This method not only allows us to optimize the data used for PCA and ICA but also provides a robust framework for improving the classifier’s accuracy. Furthermore, these techniques could be refined and additional machine learning algorithms explored to extend the promising results we have demonstrated here.

In future work, we propose exploring using various other feature selection methods as alternatives to the Kruskal–Wallis test before applying PCA and ICA and using deep learning methods instead of an SVM.

## Figures and Tables

**Table 1:** Description of the databases.

Database	Language	Size	Classes	Features
SAVEE	English	480	7	6373
RAVDESS	English	1440	8	6373
Berlin	German	535	7	6373

**Table 2:** Descriptions of frequently used mathematical notation.

Notation	Description
Y	Label of emotions
X	Features extracted from the audio with openSMILE 3.0.2 [8]
$X_{\mathrm{norm}}$	Features normalized between 0 and 1
$X_{\mathrm{fnorm}}$	Features selected with a variance threshold of $10^{-5}$
$X_{s}$	Features selected with variance and standardized μ=0 and σ=1
$X_{\mathrm{KW}}$	Features selected by the Kruskal–Wallis test
$X_{n}$	Features with a normal distribution
$X_{\mathrm{nn}}$	Features with a non-normal distribution
$S_{\mathrm{ICA}}$	Independent components
$Z_{\mathrm{PCA}}$	Principal components
W	Estimated matrix of independent signals
$W_{P}$	Matrix of the eigenvectors used in PCA
A	Mixing matrix of independent signals
*C*	Regularization parameter for an SVM
γ	Kernel coefficient for an SVM
$\sigma_{X}$	Variance in data X

**Table 3:** Features filtered by variance and separated by distribution.

Database	Variance	Normal	Non-Normal	Kruskal–Wallis	Normal KW	Non-Normal KW
	Number of Features					
SAVEE	5881	599	5282	4382	415	3967
RAVDESS	5768	171	5597	4845	133	4712
Berlin EmoDB	5975	475	5500	5407	397	5010

**Table 4:** Matrix dimensions for each database.

	SAVEE	RAVDESS	Berlin EmoDB
$X_{S}$	480×5881	1440×5768	535×5975
$X_{\mathrm{KW}}$	480×4382	1440×4845	535×5407
$X_{n}$	480×415	1440×133	535×397
$X_{\mathrm{nn}}$	480×3967	1440×4712	535×5010

**Table 5:** Parameters for the FastICA algorithm.

Parameters	Values
Algorithm	Deflation
Whiten	Unit variance
Fun	logcosh
fun_args	`alpha’:1.0
tol	$1\times 10^{-4}$
max_iter	500
w_init	None

**Table 6:** Comparison with the proposed method based on recognition performance.

Database	Method	Classifier	Author	Split Ratio	Accuracy (%)
SAVEE	235 PCA	SVM	[14] (2019)	10-fold CV	72.39
	PCA+LDA	SVM	[12] (2018)	80-20	72.23
	**PCA+ICA**	**SVM**	**Ours**	**10-fold CV**	**75.40**
EmoDB	264 PCA	SVM	[14] (2021)	10-fold CV	81.71
	**PCA+ICA**	**SVM**	**Ours**	**10-fold CV**	**84.30**
RAVDESS	PCA	SVM	[43] (2022)	10-fold CV	42.96
	**PCA+ICA**	**SVM**	**Ours**	**10-fold CV**	**59.90**

**Table 7:** Comparison of the proposed method’s recognition performance without applying feature reduction transformation techniques.

Database	Method	Classifier	Author	Split Ratio	Accuracy (%)
SAVEE	129 feats	SVM	[14] (2019)	10-fold CV	77.92
	LLDs+VGGishs	DNN	[44] (2021)	NA	66.20
	MFMC	SVM	[45] (2021)	10-fold CV	75.63
	GWO	KNN	[46] (2023)	90-10	83.54
	**PCA+ICA**	**SVM**	**Ours**	**10-fold CV**	**75.40**
EmoDB	86 feats	SVM	[14] (2019)	10-fold CV	84.07
	MFMC	SVM	[45] (2021)	10-fold CV	81.5
	GA	SVM	[47] (2023)	5-fold CV	85.6
	Spectrogram	GRU network	[48] (2024)	leave-one-speaker-out (LOSO)	88.93
	**PCA+ICA**	**SVM**	**Ours**	**10-fold CV**	**84.30**
RAVDESS	MFMC	SVM	[45] (2023)	10-fold CV	64.31
	2D+VGG-16	DNN	[49] (2022)	80-20	81.94
	182 feats	SVM	[46] (2023)	90-10	49.65
	GWO	KNN	[46] (2023)	90-10	80.48
	**PCA+ICA**	**SVM**	**Ours**	**10-fold CV**	**59.90**

## Data Availability

The original source code presented in the study are openly available in GitHub at: https://github.com/rkingeski/pca_ica_speech_emotion, (accessed on 18 June 2024).
